# Is intimate partner violence vertically transmitted among women in sub-Saharan Africa? Evidence from demographic health surveys between 2010 and 2019

**DOI:** 10.1186/s12939-023-02074-3

**Published:** 2023-12-15

**Authors:** Oluwatobi Abel Alawode, Obasanjo Afolabi Bolarinwa, Julia Marie Hajjar, Stephen Okechukwu Chukwudeh, Sanni Yaya

**Affiliations:** 1 Department of Sociology and Criminology & Law, University of Florida, Florida, USA; 2 Department of Public Health, York St John University, London, UK; 3 Department of Demography and Population Studies, University of Witwatersrand, Johannesburg, South Africa; 4 Interdisciplinary School of Health Sciences, Faculty of Health Sciences, University of Ottawa, Ottawa, ON Canada; 5 Department of Criminology and Security Studies, Federal University Oye-Ekiti, Oye-Ekiti, Ekiti, Nigeria; 6 School of International Development and Global Studies, Faculty of Social Sciences, University of Ottawa, Ottawa, ON Canada; 7 The George Institute for Global Health, Imperial College London, London, UK

**Keywords:** Intergenerational transmission, Intimate partner Violence, Sub-Saharan Africa Vertical transmission, Women, DHS

## Abstract

**Background:**

Violence against women is a major human rights violation, and the continuous occurrence of this can have many implications for women’s social and health outcomes. The experience of violence from an intimate partner could be more intriguing, especially if such women experienced their mother’s intimate partner violence (IPV) issues. Thus, this study examined the vertical transmission of IPV among women in sub-Saharan Africa (SSA).

**Methods:**

A total of 97,542 eligible women were drawn from 27 countries in SSA using a retrospective secondary dataset from Demographic Health Surveys conducted between 2010 and 2019. Multivariable analysis was employed to determine the association between the vertical transmission of IPV from mother to daughter and the covariates associated with IPV in SSA at p < 0.05.

**Results:**

The results showed that 40% of the respondents had experienced lifetime IPV, whilst 25% of those women reported that their mothers experienced it in childhood in SSA. Country-specific variations showed the highest prevalence of IPV experienced in Sierra Leone (60%) and the lowest in Comoros (9%). Results from model 1 showed that women who reported that their mothers experienced IPV were found to be significantly more than two times more likely to have experienced any form of IPV compared to those whose mothers did not (aOR = 2.66; 95% CI: 2.59–2.74), after adjusting for cofounders in Model 2, the result still showed that women who reported that their mothers experienced IPV were found to be significantly more than two times more likely to have experienced any form of IPV compared to those whose mothers did not (aOR = 2.56; 95% CI: 2.48–2.63). On the other hand, women with higher-educated partners, women in rural areas, and those from female-headed households were less likely to experience IPV.

**Conclusion:**

This study concluded that women whose mothers experienced IPV were more likely to have experienced IPV. Our study also identified that education, rural areas, and female-headed households were protective factors against experiencing IPV. To address the groups of women at higher risk for experiencing IPV, we recommend ensuring that girls complete their education to promote greater wealth and resources.

## Background

Intimate partner violence (IPV) is the physical, sexual, psychological, mental, and social harm against a person closely related, which could be in marriage, cohabitation, or intimate relationships [1]. This could be violence against a male or female (either spouse could be a victim). There are indications that women are most likely to be victims of IPV [1], which violates their mental health and human rights. The effect of IPV on women includes depression, suicide, sexually transmitted disease, dislocation, bruises, joint pain, broken bones, loss of vision, mental stress, insecurity, and death [2, 3].

Between the years 2000 to 2018, the World Health Organisation (WHO) reported that about 30% of women had experienced IPV globally [4]. One major bane of the issue is that children who observe parental spousal violence without seeing any form of resistance from the victim(s) are likely to perceive such acts as the norm [5, 6]. Similarly, in the same year, the rate of IPV remained high at about 33% among the WHO Africa region [4], despite conscious efforts to reduce its prevalence, including the United Nations Declaration on the Elimination of Violence Against Women in 1993 [7].

IPV practice in the Africa region, including sub-Saharan Africa (SSA), is highly associated with patriarchy, which restricts women from gaining economic power and reduces their confidence, thus enabling their male counterparts to exercise comparative economic and social hegemony over them [8–10]. This power inequality becomes a tool for controlling women and depriving them of their fundamental human rights. The outcome of the power relations derived from IPV correlates with female sexual exploitation, unwanted pregnancy, and control of women’s sexual and reproductive health behavior [11]. Children also suffer from violence against their mothers, as women’s psychosocial and emotional reaction to adequately care for the well-being of their children has been dehumanised by their perpetrators [12, 13].

IPV occurs irrespective of geographical location, age, ethnicity, and socioeconomic status [14], often resulting in depression, trauma, suicide, and death [15]. However, little is known in SSA about the intrinsic social learning processes of IPV that could be vertically transmitted. This is essential as previous studies have posited that aggression and violence may be learned from parents, and male children could externalise their experience within the home to become future perpetrators of IPV [14, 16]. In addition, the characteristics of perpetrators of vertically transmitted IPV are unclear in SSA. More knowledge is needed to understand females’ social acceptance of IPV in SSA.

The prevalence of IPV has implications for achieving the Sustainable Development Goal (SDG) 5, which aims to eliminate violence against women by 2030. Previous studies have highlighted the association between IPV and child mortality in the SSA [2], consumption of alcohol, IPV in SSA [17], and child marriage and partner violence [18]. These studies stressed the dangers of not achieving social goals and SDGs due to the continued prevalence of IPV. However, these studies have neglected the influence of intergenerational social learning and women’s attitudes toward IPV in SSA. Examining the variation of IPV by cohabitation, marital status, and victim’s sociodemographics is germane to formulating adequate policy to address this harmful and pervasive issue. To the best of our knowledge, at the time of this study, no other study has addressed this issue in SSA. Thus, this study is the first to examine the vertical transmission of IPV in SSA.

The goal of this study is to examine the intergenerational transmission of intimate partner violence. Previous studies have only utilised small sample surveys and/or national surveys of each country without examining the prevalence between countries in SSA to understand the crux of the high rate of IPV in the region. The need to examine the prevalence and associated factors in SSA is pertinent to a robust knowledge of the problem. This is particularly important considering the rising cases of violence in the SSA region. In fact, the report shows that IPV is highest in SSA compared to Europe, with at least one in three females likely to experience IPV in their lifetime [19]. This study is timely in SSA, where the cycle of violence has remained considerably high over time due to underreporting, gender norms, fear of retaliation, and variation in the legal definition of IPV [20, 21]. In addition, there is an absence of comprehensive analyses of IPV across countries in SSA using large surveys. This study fills these gaps by examining the vertical transmission of IPV among women in SSA between 2010 and 2019, using data from 27 countries in SSA. Further, we explored country variation of IPV by women’s age distribution, level of education and place of residence. Using the latest datasets from SSA, the study shows the geographical heterogeneity of the prevalence of IPV in SSA.

## Data and methods

### Study design and participants

The study utilised a retrospective secondary dataset from the Demographic and Health Surveys (DHSs) collected using a cross-sectional design in 27 countries in SSA between 2010 and 2019. The DHS is well known for its adequate national representativeness and has been conducted in more than 90 low- and middle-income countries (LMICs) [22]. Questions regarding participants’ socio-demographic characteristics, maternal and child health, and other sexual and reproductive health-related indicators such as HIV and STI testing, family planning use, abortion, intimate and sexual partner violence, etc., are typically asked among women aged 15–49 using a questionnaire survey design [23].

The DHS involves a two-stage sampling procedure, which consists of a primary survey unit from which participants are randomly selected from clusters in each country included in this study [22, 23]. One ever-married woman per household, aged between 15 and 49 years from every third household selected, was eligible to participate in the domestic violence module. The participants were ever married or cohabiting. This resulted in a total sample size of 97,542, and specific countries’ sample sizes across SSA are indicated in Table 1 for this study. The survey is recognised as a high-quality secondary dataset and has been previously used to conduct studies on sexual and reproductive health in SSA [10, 24]. The DHS datasets employed in this study are publicly available on the DHS website and can be downloaded for free upon request via https://dhsprogram.com/data/available-datasets.cfm.

**Table 1:** Selected DHS Countries, survey year, sample sizes and percentage

Country	Survey Year	Sample size	Percent
Angola	2015/16	5,231	5.36
Benin	2017/18	3,781	3.88
Burundi	2016/17	6,471	6.63
Congo DR	2013/14	4,305	4.41
Cameroon	2018	3,837	3.93
Ethiopia	2016	4,122	4.23
Gabon	2012	2,355	2.41
Gambia	2013	1,487	1.52
Kenya	2014	3,318	3.40
Comoros	2012	1,849	1.90
Liberia	2013	1,470	1.51
Madagascar	2008/09	4,361	4.47
Mali	2018	2,844	2.92
Mauritania	2019/20	2,116	2.17
Malawi	2015/16	4,250	4.36
Mozambique	2011	4,215	4.32
Nigeria	2018	5,950	6.10
Namibia	2013	796	0.82
Rwanda	2014/15	1,572	1.61
Sierra Leone	2013	3,261	3.34
Senegal	2010	1,251	1.28
Chad	2014/15	2,730	2.80
Togo	2013	4,515	4.63
Tanzania	2015/16	5,915	6.06
Uganda	2016	5,627	5.77
Zambia	2018	5,450	5.59
Zimbabwe	2015	4,463	4.57

Source: authors

### Study variables

#### Outcome variable

The outcome variable of this study is the experience of any form of IPV, which we developed from a series of questions asked to the female respondents to determine their experience of physical and emotional violence. In the DHS, the questionnaire first collected information about emotional violence and then moved to physical forms of violence. For emotional violence, the questions asked were: Does (did) your (last) husband ever (1) Say or do something to humiliate you in front of others? (2) Threaten to hurt or harm you or someone close to you? (3) Insult you or make you feel bad about yourself? The responses to these questions were “Yes” and “No”. Women who answered “Yes” to any of these questions were labelled as having “experienced emotional violence” coded as 1 and 0 otherwise.

For physical violence, the questions used for recording information related to physical violence were: Does (did) your (last) husband ever (1) Push you, shake you, or throw something at you? (2) Slap you? (3) Twist your arm or pull your hair? (4) Punch you with his fist or with something that could hurt you? (5) Kick you, drag you, or beat you up? (6) Try to choke you or burn you on purpose? (7) Threaten or attack you with a knife, gun, or any other weapon? Similar to what was done above, if the response options were “Yes” and “No”. Women who answered “Yes” to any one of these questions were labelled as having “experienced physical violence” and coded 1 and 0 otherwise. Subsequently, these variables were summed together to develop the IPV variable. Women who answered “Yes” to any of the above-mentioned ten questions were labelled as having “experienced IPV” and coded as “1”, and those who answered “No” to.

all questions were labelled as “did not experience IPV” and coded as “0” [25].

#### Explanatory variables

Similar to previous studies, the key explanatory variable in this study is the respondent’s mother’s experience with IPV. The survey asked the respondents: “whether the respondent’s father ever hit her mother”. The response to this question were “Yes”, coded as 1, and “No” was coded as 2 [14, 25].

### Covariates

The study covariates in the regression analysis were selected for their bivariate association with the outcome variable and the literature [26, 27]. These include age, age at first marriage, women’s highest level of education, partner’s highest level of education, type of place of residence, parity, type of marriage, work status, level of exposure to mass media, household wealth index, and the sex of the household head. The age of the respondents was categorised into 15–24, 25–34, and 35+; Age at first marriage had two categories, including less than 18 years and married at 18 years or higher. The level of education for the women and their partners was categorised as no education, primary education, secondary and higher education; the type of place was categorised into urban and rural. Another important covariate included in this study is parity, which had three categories: 0, 1–4, and 5+; type of marriage was categorised into monogamy and polygamy; Work status was categorised into working and not working; Level of mass media exposure was categorised into no exposure, low exposure, and high exposure. The categories of the household wealth index were based on DHS, which was measured based on the items available in each household. Principal component analysis was then used to group the available items into ‘poorest’, ‘poorer’, ‘middle’, ‘richer’, and ‘richest’, which represented the household wealth index, and this same measure was adopted in this current study. Lastly, the sex of the household head was categorised into either male or female [10, 17, 18].

### Statistical analyses

**Fig. 1:**
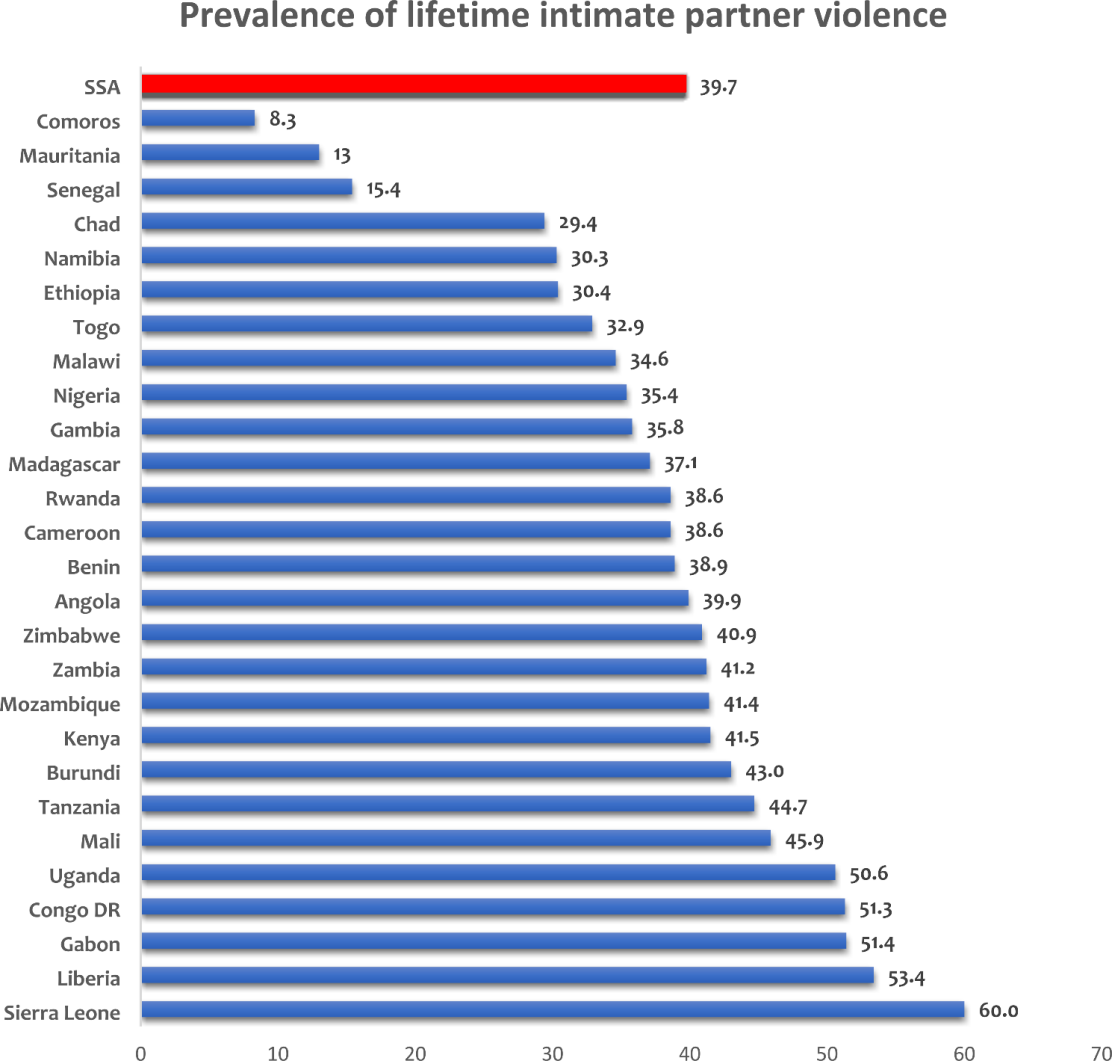
Country-Level Prevalence of Lifetime Experience of Intimate Partner Violence

The study employed three steps to analyse the included datasets in this study using the STATA 17.0 version. Table 1 was computed by the authors based on the eligible women’s sample size from each country, and these sample sizes were used to generate represented percentages for each country. Furthermore, all generated weighted datasets in the 27 countries were merged to first generate the prevalence of any form of IPV testing among women in all 27 SSA countries in the study. This was represented using a bar graph (Fig. 1). Secondly, IPV was cross-tabulated with respondents’ childhood experience of their mother’s IPV and other variables in the study, and the results were presented in Table 2, showing percentages, frequency distributions, and chi-square (*X*^2^) results. Lastly, three binary logistic regression models were fitted at the multivariate level. “Model 1” shows the unadjusted odd ratios (cOR) relationship between respondents’ childhood experience of their mother’s IPV and their own experience of any form of IPV. In “Model 2”, age, the highest level of education of both the women and their partners, age at first marriage, household wealth index, the type of place of residence, type of marriage, work status, level of exposure to mass media, and sex of the head of household were added as covariates to show an adjusted relationship (aOR) between respondents’ childhood experience of their mother’s IPV and their own experience of any form of IPV, while in model 3 we included the interaction term of respondents’ childhood experience of their mother’s IPV and their country of residence The confidence intervals were also presented across all models, and statistical significance was set at p < 0.001 and p < 0.005. The analysis accounted for non-response and under-sampling by applying the survey sample weight.

## Results

Figure 1 shows country-specific variations; the highest prevalence of any form of IPV experience is found in Sierra Leone (60.0%), and the lowest is in Comoros (8.3%). Other countries with high prevalence include Liberia (53.4%), Congo DR (51.3%), and Gabon (51.4%) (Fig. 1).

The analysis also showed that 27% of the respondents reported that their mothers experienced IPV in childhood. About one-third of all respondents were 35 and older. More than half of the respondents married at 18 years and above (56.5%). It was also found that about a quarter of the women completed secondary school (25.6%), and 35.3% reported that their partners completed primary education. In terms of exposure to mass media, 44.2% had low exposure. In addition, 18.9% of the respondents were from the richest households. Finally,83.7% of respondents reported that males headed their households (Table 2).

The bivariate analysis employing the chi-square found that a bivariate association existed between childhood experience of IPV, demographic, socioeconomic characteristics, and current experience of any form of IPV (p < 0.001). It was found that 56.5% of those who reported that their mothers experienced IPV reported lifetime experience of IPV. The analysis also revealed that the highest percentage of IPV experienced by age is among women 25–34 (40.5%), while the higher percentage of those who have experienced IPV married at less than 18 years of age (41.4%). A higher percentage of IPV was also experienced among women with low exposure to mass media (41.3%). In addition, 42.1% of women in the poorest household index reported experiencing IPV. Finally, 40.2% of women living in male-headed households reported experiencing IPV (Table 2).

**Table 2:** Descriptive Characteristics of study respondents and distribution of lifetime experience of intimate partner violence by Intergenerational Transfer of Intimate Partner Violence and other selected Characteristics

	n = 97,542	Lifetime experience of Intimate Partner Violence		
Variables	Freq. [%]	No[%]	Yes [%]	*P*-Value
**Intergenerational Transfer of Intimate Partner Violence (Mother also experienced intimate partner violence)**				< 0.001
No	71,165 [72.9]	47,533 [67.2]	23,175 [32.8]	
Yes	26,377 [27.0]	11,524 [43.5]	14,963 [56.5]	
**Age**				< 0.001
15–24	23,751 [24.4]	15,248 [63.7]	8,673 [36.3]	
25–34	41,688 [42.7]	24,334 [59.5]	16,564 [40.5]	
35+	32,101 [32.9]	19,475 [60.2]	12,901 [39.9]	
**Age at marriage**				< 0.001
< 18	42,418 [43.5]	275,194 [58.9]	17,612 [41.4]	
18+	55,124 [56.5]	33,863 [62.3]	20,526 [38.7]	
**Highest Level of Education**				< 0.001
No Education	30,700 [31.5]	16,655 [62.4]	11,844 [37.6]	
Primary	37,714 [38.7]	21,609 [57.4]	16,031 [42.6]	
Secondary	24,988 [25.6]	15,064 [61.2]	9,296 [38.2]	
Higher	4,139 [4.2]	2,729 [73.8]	967 [26.2]	
**Partner’s Highest Level of Education**				< 0.001
No Education	24,393 [25.0]	16,172 [64.3]	8,980 [35.7]	
Primary	34,385 [35.3]	19,259 [57.0]	14,535 [43.0]	
Secondary	31,006 [31.8]	18,457 [59.7]	12,437 [40.3]	
Higher	7,757 [7.9]	5,169 [70.3]	2,186 [29.7]	
**Type of Place of Residence**				< 0.001
Urban	32,797 [33.6]	19,633 [62.6]	11,736 [37.4]	
Rural	6964,774 [66.4]	39,424 [59.9]	26,402 [40.1]	
**Parity**				< 0.001
0	6,020 [6.2]	4,445 [74.2]	1,550 [25.9]	
1–4	60,890 [62.4]	36,771 [61.4]	23,144 [38.6]	
5+	30,631 [31.4]	17,841 [57.0]	13,444 [43.0]	
**Type of Marriage**				< 0.001
Monogamy	80,841 [82.9]	49,657 [62.1]	30,366 [37.9]	
Polygamy	16,701 [17.1]	9,400 [54.7]	7,772 [45.3]	
**Work Status**				< 0.001
Not working	26,334 [27.0]	18,581 [68.5]	8,535 [1.5]	
Working	71,207 [73.0]	40,476 [57.8]	29,603 [42.2]	
**Level of Mass Media Exposure**				< 0.001
No Exposure	33,363 [34.2]	21,484 [61.7]	13,342 [38.3]	
Low Exposure	43,102 [44.2]	25,133 [58.7]	17,661 [41.3]	
High Exposure	21,076 [21.6]	12,440 [63.6]	7,135 [36.5]	
**Household Wealth Index**				< 0.001
Poorest	19,679 [20.2]	12,747 [57.8]	9,277 [42.1]	
Poorer	20,053 [20.6]	11,819 [58.5]	8,387 [41.5]	
Middle	19,772 [20.3]	11,725 [59.9]	7,847 [40.1]	
Richer	19,532 [20.0]	11,275 [61.8]	6,974 [38.2]	
Richest	18,504 [18.9]	11,491 [67.0]	5,653 [33.0]	
**Sex of Household Head**				< 0.001
Male	81,659 [83.7]	48,609 [60.3]	32,063 [40.2]	
Female	15,883 [16.3]	10,448 [63.1]	6,102 [36.9]	
**Total**	**97,542 [100%]**	**59,057 [60.8]**	**38,165 [39.2]**	

Table 3 shows the binary logistic regression of the association between intergenerational transmission of IPV and experience of any form of IPV. Two separate models were fitted. In model 1, which is the unadjusted model, women who reported that their mothers experienced IPV were found to be significantly more than two times more likely to have experienced any form of IPV compared to those whose mothers did not (OR = 2.66; 95% CI: 2.59–2.74). In Model 2, the results still showed that women who reported that their mothers experienced IPV were found to be significantly more than two times more likely to have experienced any form of IPV compared to those whose mothers did not (aOR = 2.40; 95% CI: 2.33–2.47). The covariates also showed significant association with experience of IPV. Older women were more likely to have experienced any form of IPV compared to younger women, and higher education for the women and their partners was associated with lower odds of experiencing any form of IPV.

A higher household wealth index category was also associated with a significantly lower likelihood of experiencing any form of IPV. The results also showed that women who married at 18 years and above, as well as women residing in rural areas, were 0.86 and 0.87 times less likely to have experienced any form of IPV than those who married at less than 18 years or resided in urban areas. Women in polygamous marriages were 1.36 times more likely to experience any form of IPV compared to women in monogamous marriages (aOR = 1.36; 95% CI: 1.31–1.41). Working women are 1.51 times more likely to experience any form of IPV than women who were not working (aOR = 1.51; 95% CI: 1.47–1.56). Finally, women from female-headed households were 0.88 times less likely to experience any form of IPV than those from male-headed households (aOR = 0.88; 95% CI: 0.84–0.91).

In model 3, the findings remained similar to what was found in model 2, even after we tested the interaction of respondents’ experience of their mother’s IPV and their country of residence to know the effect on experience of IPV. The result of the interaction term showed that women in Benin and Ethiopia who experienced their mother’s IPV are 1.32 times more likely to have experienced IPV compared to women in Angola. The odds of experiencing IPV for women who saw their mothers experience IPV in Mauritania and Chad were 2.29 and 2.46 times higher compared to such women in Angola.

**Table 3:** Binary Logistic Regression of the Relationship Between Intergenerational Transmission of Intimate Partner Violence and Lifetime Intimate Partner Violence and Controlling for respondents’ Demographic and Socioeconomic Characteristics

	Model 1	Model 2	Model 3
Any Form of Spousal Violence	cOR [95% CI]	aOR [95% CI]	aOR [95% CI]
**Intergenerational Transfer of Spousal Violence (Mother also experienced spousal violence)**			
No [ref.]			
Yes	2.66 *** [2.59–2.74]	2.56 *** [2.48–2.63]	2.06 *** [1.82–2.33]
**Age**			
15–24 [ref]			
25–34		1.24 *** [1.20–1.29]	1.26 *** [1.21–1.30]
35+		1.18 *** [ 1.13–1.22]	1.19 *** [ 1.14–1.23]
**Highest Level of Education**			
No Education [ref.]			
Primary		1.10 *** [1.06–1.14]	1.11 *** [1.06–1.15]
Secondary		1.09 *** [1.04–1.14]	1.05 *** [1.01–1.11]
Higher		0.78 *** [0.71–0.86]	0.77 *** [[0.69–0.84]
**Partner’s Highest Level of Education**			
No Education [ref.]			
Primary		1.21 *** [1.16–1.26]	1.17 *** [1.12–1.22]
Secondary		1.19 *** [1.14–1.25]	1.08 *** [1.03–1.13]
Higher		0.95 [0.88–1.02]	0.85 *** [0.79–0.92]
**Age at first marriage**			
< 18 [ref.]			
18+		0.86 *** [0.84–0.89]	0.86 *** [0.83–0.88]
**Household Wealth Index**			
Poorest [ref.]			
Poorer		0.94 ** [0.90–0.98]	0.95 *** [0.92–0.99]
Middle		0.87 *** [0.84–0.91]	0.90 *** [0.86–0.94]
Richer		0.79 *** [0.76–0.83]	0.83 *** [0.79–0.87]
Richest		0.67 *** [0.63–0.71]	0.72 *** [0.67–0.76]
**Type of Place of Residence**			
Urban [ref.]			
Rural		0.87 *** [0.84–0.90]	0.88 *** [0.85–0.91]
**Type of Marriage**			
Monogamy [ref.]			
Polygamy		1.36 *** [1.31–1.41]	1.31 *** [1.26–1.36]
**Work Status**			
Not Working [ref.]			
Working		1.51 *** [1.47–1.56]	1.35 *** [1.31–1.40]
**Level of Mass Media Exposure**			
No Exposure [ref.]			
Low Exposure		1.15 *** [1.12–1.19]	1.18 *** [1.14–1.22]
High Exposure		1.08 *** [1.03–1.13]	1.16 *** [1.11–1.22]
**Sex of Household Head**			
Male [ref.]			
Female		0.88 *** [0.84–0.91]	0.91 *** [0.88–0.95]
**Mother experienced IPV & Country**			
Yes*Benin			1.32 *** [1.03–1.70]
Yes*Burundi			0.94 [0.80–1.11]
Yes*Congo DR			1.04 [0.87–1.23]
Yes*Cameroon			1.42 *** [1.16–1.73]
Yes*Ethiopia			1.32*** [1.09–1.61]
Yes*Gabon			1.15 [0.94–1.41]
Yes*Gambia			1.46 *** [1.06–2.00]
Yes*Kenya			1.04 [0.87–1.25]
Yes*Comoros			0.97 [0.53–1.75]
Yes*Liberia			0.93 [0.72–1.19]
Yes*Madagascar			1.56 *** [1.25–1.93]
Yes*Mali			1.66 *** [1.23–2.24]
Yes*Mauritania			2.29 *** [1.30–4.03]
Yes*Malawi			0.88 [0.73–1.06]
Yes*Mozambique			1.22 *** [1.01–1.48]
Yes*Nigeria			1.83 *** [1.49–2.24]
Yes*Namibia			1.15 [0.84–1.59]
Yes*Rwanda			0.97 [0.76–1.24]
Yes*Sierra Leone			1.23 [0.99–1.51]
Yes*Senegal			1.99 *** [1.01–3.91]
Yes*Chad			2.46 *** [1.93–3.15]
Yes*Togo			1.24 ***[1.02–1.51]
Yes*Tanzania			1.44*** [1.22–1.70]
Yes*Uganda			1.06 [0.90–1.25]
Yes*Zambia			1.12 [0.94–1.32]
Yes*Zimbabwe			0.97 [0.81–1.16]

CI = Confidence Interval; cOR = Crude Odds Ratio; aOR = Adjusted Odds Ratio; ref = Reference; ***=p < 0.05

Model 1 showed the uncontrolled or unadjusted relationship between the Intergenerational Transfer of Intimate Partner Violence and the lifetime experience of intimate partner violence

Model 2 controlled for covariates such as age, the highest level of education of both the women and their partners, age at first marriage, household wealth index, the type of place of residence, type of marriage, work status, level of exposure to mass media, sex of the head of household, and country of residence

Model 3 controlled for covariates such as age, the highest level of education of both the women and their partners, age at first marriage, household wealth index, the type of place of residence, type of marriage, work status, level of exposure to mass media, sex of the head of household, and interaction of Intergenerational Transfer of Intimate Partner Violence and country of residence

## Discussion

This study examined whether IPV was vertically transmitted among women in SSA using secondary DHS datasets in 27 countries from 2010 to 2019. Factors significantly associated with women experiencing IPV were having mothers who experienced IPV. If women were age 25 or older, in polygamous marriages, if women were working, and if women had low or high exposure to mass media. Factors significantly associated with a reduced likelihood of women experiencing IPV were the completion of higher education for both spouses, marrying at age 18 or over, belonging to the richest household wealth index category, residing in rural areas, and living in female-headed households. Experience of IPV has many deleterious consequences on maternal-child health, including a greater risk of pregnancy termination [18], higher risk of HIV [28], higher rates of child mortality [2], and child marriage [29]. Exploring vertical transmission of IPV is thus vital to identifying and addressing the root causes driving the cycle of violence across generations.

Women in our study were over twice as likely to experience IPV if their mothers had experienced IPV and 1.7 times more likely if their mother’s history with IPV was unknown compared to women whose mothers did not experience IPV. Cultural attitudes and acceptance towards IPV continue to drive the cycle and prevent its mitigation [30]. In a study in Pakistan, vertical transmission of IPV was reportedly driven by girls’ learned behaviour and acceptance of violence observed in the home, perpetuating the cycle in their own lives [31]. Social support is a pivotal strategy to better empower and support women experiencing IPV. In Tanzania, family support was identified as a key protective factor to mitigate IPV in women receiving antenatal care in Moshi Municipality [11]. Further, the societal structure can dictate the level of acceptance of IPV. For instance, IPV may be less accepted in matrilineal communities compared to patrilineal communities, as women likely reside closer to their own families and have subsequent support systems in place [30].

Our study illustrates that an intergenerational cycle of violence is evident. A leading theory underpinning this phenomenon is the intergenerational transmission of violence theory, which posits that children and adolescents adopt violence as a learned behaviour through exposure to domestic violence in the household and subsequently use violence in the context of intimate partner relationships later in life [32]. In the context of our study, it is important to note that women who worked remained at higher risk of experiencing IPV despite traditionally protective factors such as likely being more educated and generating their income. This illuminates the continued role of patriarchal oppression of women in this region and the rigid gender roles that must be addressed in order to mitigate IPV in SSA.

The literature has reported similar findings to our study pertaining to country-level variations in IPV prevalence, with a study that explored sexual autonomy and IPV also reporting that the highest prevalence of IPV occurred in Sierra Leone and the lowest prevalence of IPV occurred in Comoros [33]. Higher rates of IPV have also been reported in conflict regions, including DRC, Uganda, and Sierra Leone [34], as well as in Liberia [35], similar to the findings of our study. The association between conflict settings and higher rates of IPV may explain why Sierra Leone, Liberia and DRC had the highest rates of IPV in our study, as conflict can compromise the social protections in place that help to protect women against violence [33, 34].

IPV is widely accepted in countries throughout SSA when women do not adhere to traditional gender norms [36]. This aligns with the findings of our study, which indicated that working women had a higher chance of experiencing IPV compared to non-working women. Furthermore, social and cultural norms and the patriarchal structures prevalent in SSA continue to drive IPV by promoting its normalisation and acceptance, as reported in Uganda [37] and Nigeria [38]. Kenya did not have a significantly higher or lower rate of IPV compared to Angola in our study, and this may be due to the implementation of programs that reduced IPV by focusing on community engagement in rural regions [39]. Additionally, the Men Engage Kenya Network (Menken) initiative likely also played an important role. This NGO focused on reducing gender-based violence and preventing HIV in the country and prompted the creation of the 2013 Protection Against Domestic Violence Bill. It will, therefore, be vital to ensure a focus on creating social and structural-level contingency plans to ensure that women remain supported in conflict settings. In addition, it will be important to create and implement programs that engage communities in order to foster discussions around gender roles and begin to shift attitudes and perceptions around gender norms to reduce IPV.

### Implications for research and policy

Findings from our study further the understanding of factors associated with vertical transmission of IPV in SSA with important policy implications. Low education level is a root driver of IPV; thus, we recommend focusing on initiatives that increase girls’ educational attainment. Ensuring access to complete education is an important upstream strategy that can provide more opportunities for employment, subsequent low income, and loss of personal and financial autonomy that contribute to gaps in relationship power dynamics.

### Strengths and limitations

This study analysed secondary data from the DHS surveys in 27 countries in SSA and included a total sample size of 104,959 participants. The DHS surveys are high-quality, nationally representative, capture health information from many participants, and have been used in prior studies on sexual and reproductive health in SSA. Although a large number of countries in SSA were included in the study, it was not possible to analyse all countries in the region. DHS data collected via interviews with respondents may have a limitation of recall bias.

## Conclusion and recommendations

This study concluded that women whose mothers experienced IPV, women of older age, women in polygamous marriages, and working women were more likely to have experienced IPV. Our study also identified that education, belonging to the richest household wealth index category, residing in rural areas, and living in female-headed households were protective factors against experiencing IPV. To address the groups of women at higher risk for experiencing IPV, we recommend ensuring that girls complete their education to promote greater wealth and resources. This strengthens female autonomy, empowers women to achieve financial independence and closes the gap in power dynamics in a relationship, making it easier to regain control and leave an abusive relationship. We recommend implementing community-level educational workshops tailored to partners to enhance dialogue and education about IPV, provide supportive networks and resources for women, and gradually shift cultural attitudes and norms around IPV. This study is the first to consider intergenerational social learning and women’s attitudes toward IPV in SSA. It is a valuable step towards informing sustainable policy change that adequately addresses the pervasive issues that continue to drive IPV across generations.

## Data Availability

The datasets utilised in this study can be accessed at https://dhsprogram.com/data/available-datasets.cfm.

## Abbreviations

IPV: Intimate partner violence
SSA: Sub-Saharan Africa
SDG: Sustainable Development Goal
DHS: Demographic and Health Survey
LMICs: Low-and middle-income countries
cOR: Unadjusted odds ratio
aOR: Adjusted odds ratio
